# Pulmonary hypertension in late-onset Methylmalonic Aciduria and Homocystinemia: a case report

**DOI:** 10.1186/s12887-020-02130-9

**Published:** 2020-05-22

**Authors:** Ling-yi Wen, Ying-kun Guo, Xiao-qing Shi

**Affiliations:** 1 Department of Radiology, Key Laboratory of Birth Defects and Related Diseases of Women and Children of Ministry of Education, West China Second University Hospital, Sichuan University, Chengdu, Sichuan China; 2 Department of Cardiology, Key Laboratory of Birth Defects and Related Diseases of Women and Children of Ministry of Education, West China Second University Hospital, Sichuan University, 20# Section 3 South Renmin Road, Chengdu, Sichuan China

**Keywords:** Cobalamin C deficiency, Pulmonary hypertension, Methylmalonic aciduria, Homocystinemia

## Abstract

**Background:**

Methylmalonic Aciduria and Homocystinemia, cobalamin C (cblC) is an inherited disease of vitamin B_12_ metabolism with a wide spectrum of clinical manifestations. cblC presenting with pulmonary hypertension (PH) as leading sympotom is rare and easily misdiagnosed because of limited awareness. Timely diagnosis is crucial by the relentless progression without appropriate treatment.

**Case presentation:**

We reported a 12-year-old girl with a 3-year history of progressively reduced activity tolerance and a 3-month history of orthopnea. Metabolic testing revealed increased levels of plasma homocysteine and urine methylmalonic acid. cblC deficiency was subsequently confirmed by genetic testing. The patient was treated with hydroxocobalamin, betaine, folinic acid and levocarnitine for cblC disease. Sildenafil, bosentan, spironolactone and hydrochlorothiazide was administrated for PH and right heart failure. At 3-month follow-up, she had an apparent resolution of dyspnea and cyanosis. Metabolic abnormalities resolved the decrease of plasma homocysteine and urine methylmalonic acid. A right heart catheterization showed a reduced pulmonary pressure.

**Conclusions:**

This case emphasizes the importance of an early diagnosis and initiation of treatment for cblC deficiency. Unexplained PH in children and young adults should prompt metabolic screening for the differential diagnosis.

## Background

Methylmalonic aciduria and homocystinemia, cobalamin C (cblC) type is a rare genetic metabolic disease, with an estimated prevalence of one per 11,160 to 250,000 population worldwide [1]. The clinical manifestations and age of presentation of cblC are heterogeneous. Late-onset cblC deficiency refers patients that have symptoms after 4 years of age [2]. They usually present with a milder phenotype typically including neurologic and developmental abnormalities. Pulmonary hypertension (PH) in patients with cblC deficiency is rare but lethal complications. To our knowledge, only 4 late-onset cblC deficiency patients have been reported to have PH, [3–6] and none of them were first-presentation in PH patients. Here, we report a 12-year-old girl with cblC deficiency, who presented with PH as her first symptom.

## Case presentation

A 12-year-old girl with a 3-year history of progressively reduced activity tolerance and a 3-month history of orthopnea presented to the emergency department. On physical examination, her blood pressure was 106/59 mmHg, heart rate was regular at 120 beats per minute, respiratory rate was 35 breaths per minute, and oxygen saturation level was 88% under nasal oxygen inhalation. The patient was afebrile. She had no sign of headache, convulsions, epilepsy and changes in urine volume. Also, neruologic, psychiatric and ophthalmologic evaluation showed no positive signs. Physical examination revealed accentuated P2 cardiac sounds, a grade II systolic and diastolic murmur along the left sternal border, mild bibasilar crackles, and lower extremity edema. She had no significant medical, surgical or family history and no history of alcohol or drug use. She was born at term by natural birth. Birth parameters were normal. Her baseline weight was 40 kg (BMI, 15.4 kg/m^2^).

**Fig. 1:**
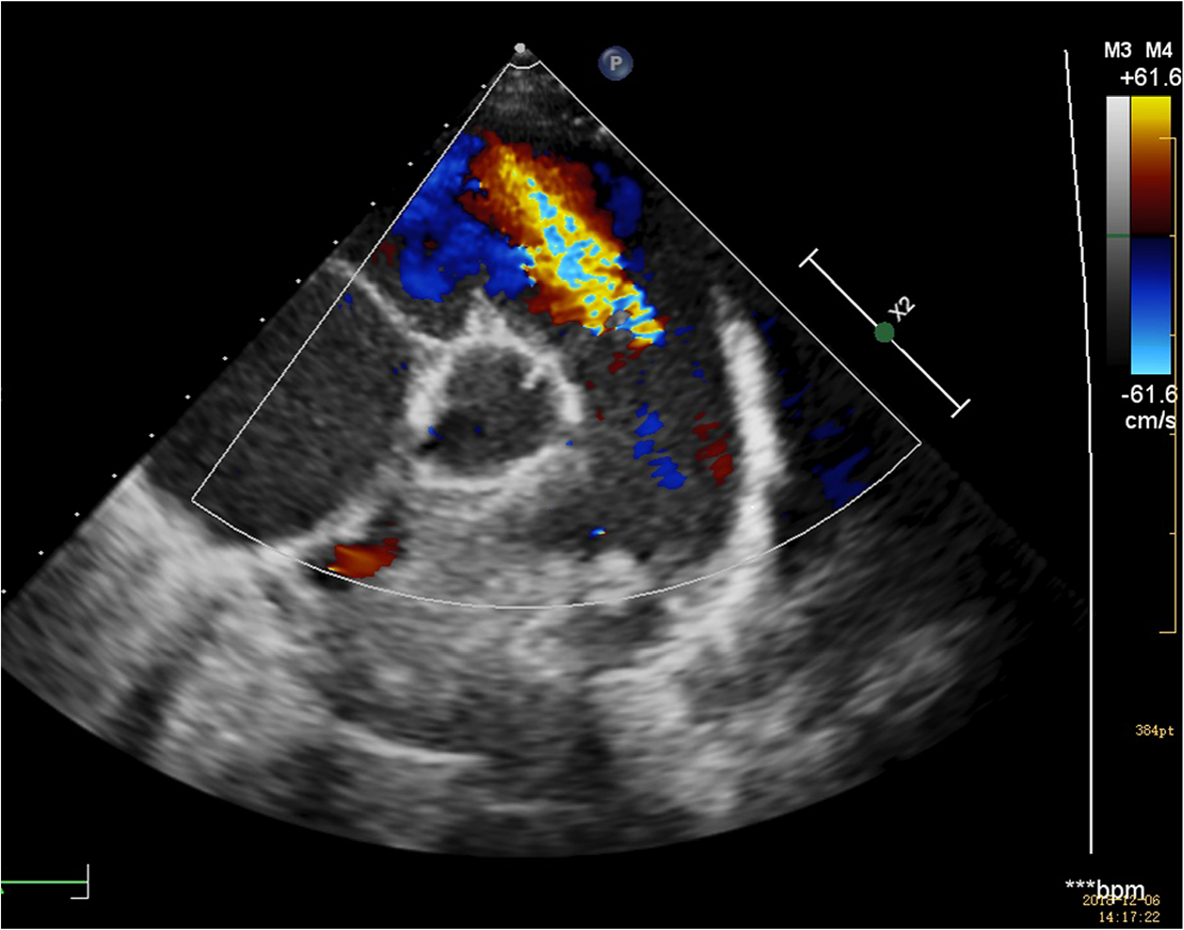
Transthoracic echocardiogram demonstrates widened pulmonary artery trunk and pulmonary regurgitation

**Fig. 2:**
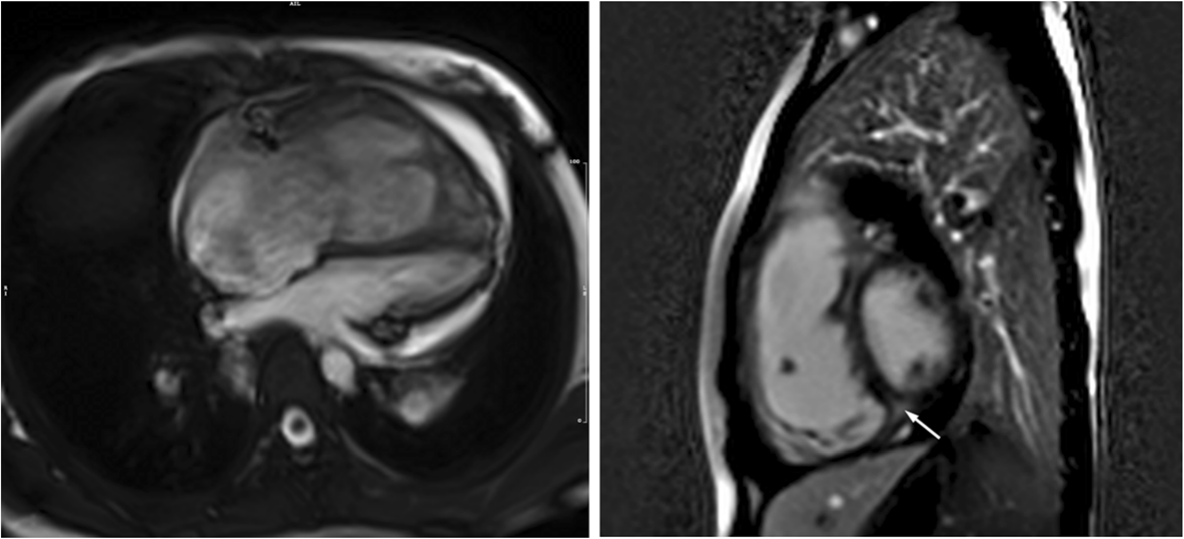
Cardiac magnetic resonance imaging confirms dilated right ventricle with late enhancement at the inferior right ventricular insertion point (arrow)


A 12-lead electrocardiogram showed T wave changes in lead II, III, aVF, V3–6 and ST segment depression. Laboratory test results are summarized in Table 1. Abnormal laboratory findings included elevated N-terminal-pro B-type natriuretic peptide, elevated plasma uric acid, elevated creatinine and decreased red cell count. Blood electrolytes and liver function were within reference limits. Urine dipstick showed no microscopic haematuria and proteinuria. A transthoracic echocardiogram revealed dilated right ventricle (the inter-ventricular measurement of right ventricle is 48 mm), widened pulmonary artery trunk (immediately above the valve, 33 mm), tricuspid regurgitation (mild), and pulmonary regurgitation (mild–moderate) (Fig. 1). Cardiac magnetic resonance imaging confirmed impaired biventricular function (right ventricular ejection fraction: 15.6% and left ventricular ejection fraction: 38.9%) with late enhancement at the inferior right ventricular insertion point (Fig. 2). Cardiac catheterization showed a mean pulmonary artery pressure of 55 mmHg, pulmonary artery wedge pressure of 30 mmHg, pulmonary vascular resistance of 8.7 woods units and the withered tree sign. An additional movie file shows this in more detail [see Additional file 1].

**Table 1:** Laboratory test results of first admission

Test name	Result	Normal range
N-terminal-pro B-type natriuretic peptide (pg/mL)	3879.2	0–100
Troponin (ug/L)	0.045	0–0.06
Plasma uric acid (umol/L)	510	184–464
Plasma creatinine (umol/L)	82	24.8–70.4
Plasma urea (mmol/L)	8.25	3.2–8.2
Serum platelet count (10^9^/L)	220	100–450
Red cell count (10^12^/L)	2.5	3.82–5.5
Hemogloblin (g/L)	110	110–146
Serum iron (umol/L)	3.9	9.0–30.4
Serum VB_12_ (pmol/L)	389.51	182–672
Serum folic acid (nmol/L)	31.41	> 12.19

**Additional file 1.** Video illustrates the withered tree sign. (AVI 3465 kb)


The main features of the patient were unexplained PH, right-sided heart failure, and impaired renal function. Renal metabolic function tests showed elevated plasma homocysteine (155.8 μmol/L, 5–15), which suggested the possibility of systemic genetic disease, i.e., methylmalonic aciduria and homocystinemia, the genetic disease of vitamin B_12_ metabolism. Further metabolic testing revealed elevated urine methylmalonic acid (49.7 mmol/molCr), elevated plasma propionylcarnitine (7.3 μmol/L, 0.2–5), and decreased plasma methionine (6.2 μmol/L, 8–45). A cobalamin C (cblC) deficiency was subsequently confirmed by genetic testing of the MMACHC gene, revealing a compound heterozygous c.80A > G/c.609G > A genotype.

The patient was treated with hydroxocobalamin (1 mg, intramuscular, q.d.), betaine (1000 mg, peros, q.d.), folinic acid (5 mg, peros, t.i.d.), and levocarnitine (10 ml, peros, b.i.d.) for cblC disease. Sildenafil (20 mg, peros, t.i.d.), bosentan (62.5 mg, peros, b.i.d.), spironolactone (20 mg, peros, b.i.d.), and hydrochlorothiazide (25 mg, peros, b.i.d.) were administrated for PH and right-sided heart failure. At the 3-month follow-up, she had an apparent resolution of dyspnea and cyanosis. The metabolic abnormalities appeared to be resolving, as observed by a decrease in urine methylmalonic acid (6.32 mmol/molCr) and plasma homocysteine (119.8 μmol/L; 5–15)), along with a reduction in plasma uric acid (428 μmol/L) and creatinine (53 μmol/L). A second right heart catheterization showed reduced pulmonary artery pressure (52/30, 38 mmHg). Cardiac magnetic resonance imaging revealed a decrease in the size of the right ventricle and pulmonary trunk and an increase in biventricular function (right ventricular ejection fraction: 29.3%, left ventricular ejection fraction: 56.2%).

## Discussion and conclusion

Methylmalonic aciduria and homocystinemia, cblC type, is a rare genetic disease of vitamin B_12_ metabolism. It is caused by a mutation in the *MMACHC* gene and results in abnormal concentrations of downstream metabolites of the cobalamin pathway [2]. The classical biochemical pattern of this disease is the accumulation of methylmalonic acid and homocysteine, with decreased methionine production. The diagnosis of cblC disease can be challenging due to highly variable clinical manifestations and ages of presentation. The early-onset form is usually multisystemic and progressive, with severe neurologic, ocular, hematologic, renal, gastrointestinal, and cardiac symptoms during the first year after birth. The late-onset form presents after 4 years of age with a mild and limited phenotype ranging from a predominantly neurologic disorder to multisystemic disturbances [2]. Isolated PH or combined renal dysfunction is one of the rare phenotypes of the late-onset form [3–6]. The mechanism whereby *MMACHC* mutation might cause PH is unelucidated. It may be that the combined results of vasculopathy and thrombosis are triggered by endothelial dysfunction. The diagnosis of cblC disease is mainly based on biochemical parameters [2] and genetic testing. c.80A > G and c.609G > A were two common mutations in Chinese cblC type patinets and were reported to be associated with late-onset cblC disease. The three most common mutations in the MMACHC gene are c.271dupA, c.394C > T and c.331C > T [7]. Plasma homocysteine, urine organic acids and plasma acylcarnitine should be assessed when a cobalamin-related disorder is suspected [8]. VB_12_ insufficiency needs to be taken into consideration for differential diagnosis.

Pediatric PH is an important cause of morbidity and mortality in children. Data from the Tracking Outcomes and Practice in Pediatric Pulmonary Hypertension study has revealed that idiopathic or genetic PH accounted for 57% of registered pediatric PH patients, of which 43% of cases were associated with systemic disorders [9]. The reported age at diagnosis for idiopathic or genetic PH is usually younger (range: 0.9–11.1 years) than this patient [10]. Previous reports [3–6] on cblC disease with PH always exhibit renal thrombotic microangiopathy at the same time, which cause clinicians’ attention to systemic metabolic desease. This case enlarges the clinical spectrum of late-onset cblC deficiency. Further work is needed to understand the prevalence of CblC in children and young adults with idiopathic/unexplained PH.

Therapeutic goals are to improve clinical symptoms, normalized methionine, methylmalonic acid and homocysteine once diagnosed. Hydroxocobalamin and betaine treatment should be initiated as soon as there is a suspicion of cblC disease, and targeted PH therapy has improved survival in idiopathic and genetic PH [2, 11]. Conventional therapies for heart failure are also utilized for the treatment of children with right ventricular failure.

In conclusion, cblC deficiency presenting with PH as leading sympotom is rare and easily misdiagnosed. The diagnosis of cblC deficiency is mainly based on biochemical parameters and genetic testing. For children and young adults with unexplained PH should prompt metabolic screening, plasma homocysteine, urine organic acids and plasma acylcarnitines testing and early treatment for cblC deficiency if diagnosed. Early recognition and timely treatment may beneficially affect the course of cblC deficiency.

## Data Availability

The datasets used during the current study are included in the manuscript.

## Abbreviations

cblC: Cobalamin C
PH: Pulmonary hypertension
